# Multi-Damage Healing Ability of Modified Bitumen with Waste Plastics Based on Rheological Property

**DOI:** 10.3390/ma18163827

**Published:** 2025-08-15

**Authors:** Mingxia Li, Yiming Fang, Lingjun Liu, Qipeng Zhu

**Affiliations:** 1 School of Intelligent Construction and Civil Engineering, Luoyang Institute of Science and Technology, Luoyang 471023, China; 15006087350@163.com (Y.F.); 18985535622@163.com (L.L.); 15156918149@163.com (Q.Z.); 2 Henan Key Laboratory of Green Building Materials Manufacturing and Intelligent Equipment, Luoyang 471023, China

**Keywords:** waste plastic, bitumen, rheological property, self-healing, multiple damages

## Abstract

To explore the influence of waste plastics on the self-healing ability of bitumen, the healing effect of multiple damages, and enlarge the utility of waste plastic in pavement through dynamic shear rheology (DSR) tests, multiple repeated loading tests with fatigue–healing–fatigue as the basic cycle were conducted on modified bitumen samples containing five types of waste plastics (PET, HDPE, PP, PS, and PVC) with different dosages. The damage healing ability of bitumen of the same waste plastic with different dosage ratios and the same dosage of different waste plastics under the same healing time, loading strain, and damage degree through single and multiple loading were explored and analyzed. The results show that based on the three sets of data of the complex shear modulus, phase angle, and fatigue factor, the PS and PVC-modified bitumen have a better recovery performance than that of the other three types of modified bitumen, and the latter also has the best fatigue resistance property. To maximize the improvement effect on the healing index of bitumen, the recommended optimal dosages of PET, HDPE, PP, PS, and PVC are 2%, 2%, 2%, 6%, and 4%, respectively. PS has the best promoting effect on the damage healing ability of bitumen after undergoing multiple damages, while PET has the worst improvement effect. The findings can provide theoretical support and guidance for the wide application of waste plastic-modified bitumen pavement.

## 1. Introduction

Plastics have substantial advantages over many other materials such as cost-effectiveness, light weight, durability and ease of processing, and have therefore been widely used in various fields [1,2]. However, the used waste plastic products have the characteristics of a large quantity, wide distribution, and are difficult to recycle, forming the “white pollution” problem that is very concerning throughout the world. It is not only polluting the environment and endangering health but also occupying valuable land resources. Sinopec and Tongji University jointly launched the “2021–2030 China Express delivery industry green packaging carbon emission reduction Potential research Report” and pointed out that with the rapid development of China’s express delivery industry, the resulting cumulative carbon emissions of disposable plastic bags are expected to reach 59.61 million tons [3]. Plastic products are also one of the most important materials in the modern chemical industry. Low-density polyethylene (LDPE) [4], high-density polyethylene (HDPE) [5,6], and polystyrene (PS) [7] are the main raw materials for single-use plastic products. While recyclable plastic, such as beverage bottles and food packaging boxes and fibers, are mostly made from polyethylene terephthalate (PET) [8], polypropylene (PP) [9], polyvinyl chloride (PVC) [10,11], etc. Based on evidence from the previous literature, the addition of waste plastics as modifiers in bitumen production can not only promote bitumen properties compared to the original polymer but greatly decrease the construction costs and avoid extra environmental pollution [8,12,13]. Therefore, many scholars have begun to study the properties of different types of waste plastic-modified bitumen.

It is proven that waste HDPE-modified bitumen has a higher viscosity, stiffness, and better moisture resistance [14]. Whereas Costa et al. pointed out that HDPE-modified bitumen leads to a worse resilience and creep recovery than that of SBS-modified bitumen. The reuse of waste plastics to modify bitumen pavement is more and more noticeable in Asia [15]. Tomasz M. Majka et.al explored the possibilities of recycled poly (ethylene terephthalate) (RPET), which was obtained from storage points located in Lesser Poland. By using thermogravimetric analysis (TG), differential scanning calorimetry (DSC), and Fourier transform infrared spectroscopy (FTIR), the results have shown that bitumen pavement modified by recyclate and mineral filler has a significant impact on its performance properties [16]. Further, the results obtained from the literature [5,6,17] show that the LDPE additive is more consistent with bitumen compared with HDPE due to its weak intermolecular force. However, the difference in the influence of LDPE and HDPE on the rheological properties of bitumen was not proven.

In addition, some scholars have indicated that the size and content of PET had a more significant influence on the PI (penetration index) and softening point than the blending temperature. The penetration and ductility values reduced from 96 to 85 mm and 100 to 78 cm, respectively, with an increasing size of PET particles from 75 to 150 μm and the content from 0% to 10% in bitumen [18]. Based on a frequency scanning test and multi-stress creep recovery test, Zhou Chao et al. proved that the rubber/plastic ratio of SBR/PP particles prepared by blending styrene butadiene rubber powder with waste polypropylene particles has a significant influence on the rheological properties and compatibility of bitumen. When 1.5% compatibilizers and 0.5% antioxidants are added, the blended additives are dispersed more evenly in the bitumen phase, resulting in a more desirable micro-structure. Moreover, the elastic recovery rate of bitumen can be increased by 38%, and the non-recoverable creep compliance and softening point can be reduced by 54% and 36%, respectively [19,20]. As a warm mix additive (WMa), the waste polypropylene plastic (WPP) was used to prepare wax base-modified bitumen. And its penetration, softening point, ductility, and rotational viscosity of modified bitumen were tested. The results proved that the sample produced at 380 °C and 1.0 MPa can increase the penetration of bitumen by 61% and reduce the viscosity (135 °C) of bitumen by 48.6%. Furthermore, the modified bitumen shows favorable elasticity, rutting resistance, and adhesion properties [21]. However, recycled PP can reduce the ductility of modified bitumen and lead to a weakening resistance to fatigue cracking [21]. Some research indicated that the ductility will reduce by around 20% when the addition of waste PP in bitumen is 5% [22]. Thus, Ahmedzade stated the viscosity of waste PP-modified bitumen should be improved when used in high-temperature and high-humidity areas [23]. LDPE and PS modifiers can effectively improve the high temperature performance of bitumen. With the increase in modifier content, the complex modulus and rutting factors of LDPE and PS polymer-modified bitumen increase continuously. LDPE and PS can weaken the viscosity and enhance the elasticity of polymer-modified bitumen. The phase angle of polymer-modified bitumen decreases with the increase in modifier content [24]. Whereas in colder areas, waste PS is not helpful for cracking resistance, having undergone a drying process when mixed with bitumen mixtures [7]. And some test results reveal that a PS-modified bitumen mixture shows a relatively lower elastic performance than that of bitumen mixtures modified by PE, PP, and rubber [25]. The existence of PS can also improve the bitumen’s stiffness and make the rutting resistance better [20,26]. But more attention should be paid to the harmful items released at 70 °C. The research pointed out that the role of PVC powder improves the molecular structure of the recycled bitumen so that the recycled bitumen can effectively resist high-temperature permanent deformation [27]. Other work on the use of plastic waste (such as PP, PS, LDPE, HDPE, and their mixtures) carried out detailed tests with pen-graded bitumen 50/70. With the BBR rheometer, the degree of aging mixtures with the additives is much lower than that of base bitumen [28]. Recent studies revealed that PVC-modified bitumen shows a better rutting resistance because of the viscosity and stiffness increased with PVC [29]. Ziari et al. also proved that it can promote fatigue resistances, except for thermal cracking resistance [30].

Overall, the existing references focus on the detailed experimental studies and data comparisons of the penetration index, softening point, ductility, stiffness, and rheological properties, compatibility, adhesion properties, cracking resistance, moisture resistance, elasticity, and high temperature of PET, HDPE, PP, PS, and PVC-modified bitumen. However, most of those just researched the secondary utilization of waste plastic particles and do not involve research on the damage repair ability of modified bitumen after its application in pavement. Meanwhile, the different forms (granular, powdered, or liquid) and dosages of waste plastics in bitumen will directly affect their ability to modify bitumen. Therefore, this paper conducts a further experimental verification of the influence of waste plastic powder on the rheological properties of bitumen. It compares and analyzes the damage repair ability of modified bitumen by using the healing index parameter, with the aim of providing theoretical support and guidance for the wide application of waste plastic-modified bitumen pavement.

## 2. Materials and Methods

### 2.1. Raw Materials

The bitumen used is 70 #, which is taken from the bitumen mixing plant Henan Liujian Construction Group (Luoyang, China). Its technical indicators are shown in Table 1. The plastic powder additives are purchased from Dongguan Huachuang Plastic Raw Materials Trading Company (Dongguan, China), and PET, HDPE, PP, PS, and PVC powders are made from mineral water bottles, food packaging bags, microwave-heated lunch boxes, bowl-shaped instant noodle boxes, and agricultural plastic films, respectively. Their densities are shown in Table 2.

### 2.2. Rheological Test and Healing Test

The rheological test and self-healing test of modified bitumen were carried out by using a DHR-2 dynamic shear rheometer of Discovery series developed by the TA Company (New Castle, DE, USA). The former was conducted by the strain loading mode to obtain the complex shear modulus G*, phase angle δ, and |G*|·sinδ. The loading parameters are the same as those of the healing test. The healing test mode adopts the first fatigue loading to stop loading when the damage of the initial modulus reaches the set value and then stops loading for a period of time (healing time), and then carries out the second fatigue loading (the same loading–stopping condition as the first time), and then carries out the second static loading, the third static loading, and the fourth loading, respectively. Finally, the experimental results of each sample under different static periods and multiple repeated loading conditions were obtained, namely, the Time-Sweep mode of “fatigue-healing-fatigue-healing-fatigue- healing-fatigue”. The loading strain and loading frequency are 5% and 10 HZ, respectively [31,32]. The loading temperature and the static temperature are both 40 °C, and the damage degree (the attenuation of the initial modulus) is 50%. At the same time, in order to avoid the error of the test results caused by the adhesion of edge excess bitumen on the metal pressure rod, the excess bitumen on the edge of the parallel plate was removed by a scraper before the test began. There are five parallel specimens tested for each modified bitumen and base bitumen. Then the average value was calculated by the three closest data among them.

### 2.3. Healing Index

The dynamic complex shear modulus of bitumen is often used to characterize the self-healing effect of bitumen. The self-healing index of bitumen HI (healing index) is calculated as follows. Among them, G_0_ is the initial modulus before static, G_a_ is the termination modulus before static, and G_b_ is the initial modulus after static. The index calculated by the following formula (Equation (1)) is used to evaluate the healing ability of modified bitumen. HI_1_, HI_2_, and HI_3_ were the healing indices obtained after the first, second, and third resting, respectively.

(1) $$HI=\frac{G_{0}-G_{a}}{G_{b}-G_{a}}$$

## 3. Specimen Prepared

After preheating the base bitumen in an oven at 135 °C for about 3 h, adding plastic powders of different dosages in four steps for each to ensure the uniformity of the specimen, and mixing them with a stirrer at a speed of 1000 r per minute for about 20 min. During the mixing process, an electric furnace and a thermometer are used to ensure that the specimen temperature remains below the aging temperature (about 150 °C). It aims to prevent the aged specimen from affecting the experimental results or boiling over, which may cause safety hazards.

## 4. Results and Discussion

### 4.1. High-Temperature Rheological Property of Modified Bitumen with Waste Plastics

**Figure 1:**
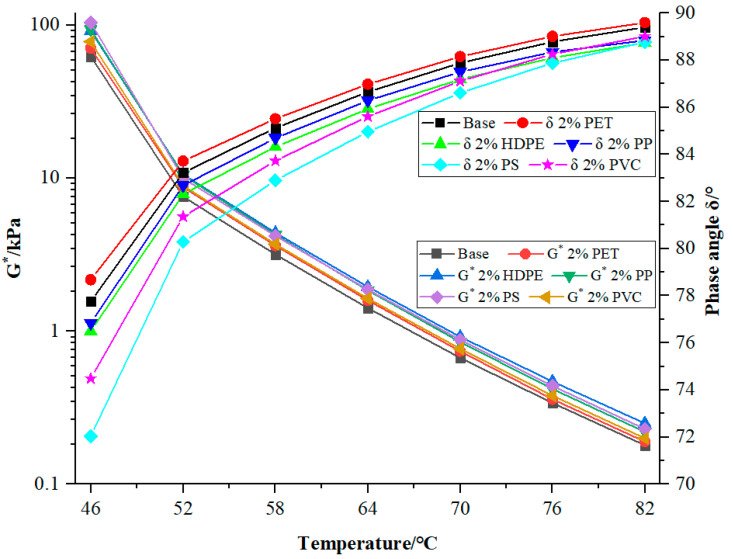
The variation diagram of the complex shear modulus G* and phase angle δ of waste plastic-modified bitumen.

Figure 1 shows the variation graphs of the complex shear modulus G* and phase angle δ of the base and modified bitumen by various waste plastics. The main curve graph of the base and modified bitumen in the figure is similar. As the temperature rises, the complex shear modulus gradually decreases, while the phase angle gradually increases. After adding different waste plastics at the same temperature, the variation rule of its complex shear modulus G* is “PS modified bitumen > PP modified bitumen > HDPE modified bitumen > PVC modified bitumen > PET modified bitumen > base bitumen”. The variation rule of its phase angle is “PET modified bitumen > base bitumen > PP modified bitumen > HDPE modified bitumen > PVC modified bitumen > PS modified bitumen”. It can be known that PS has a more obvious effect on decreasing the δ of bitumen. The phase angle characterizes the relative proportion of elastic and viscous components in bitumen material. The smaller the phase angle, the greater the elasticity of the material and the smaller its viscosity. The larger it is, the opposite is true. The phase angle of PS-modified bitumen is the lowest, which indicates that the addition of PS increases the proportion of elastic components in bitumen, and the deformation is easier to recover after unloading. The second is PVC-modified bitumen.

**Figure 2:**
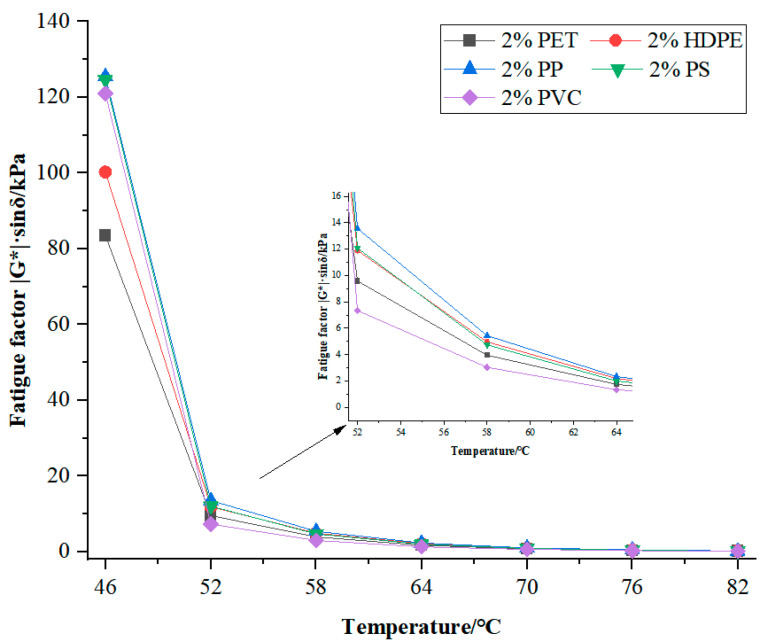
The variation diagram of the fatigue factor of waste plastic-modified bitumen.

The fatigue factor, which is the ratio of the complex shear modulus G* to the sinusoidal value of the phase angle δ (|G*|·sinδ), can be used to evaluate the fatigue resistance of bitumen, that is, its high-temperature performance. The lower the fatigue factor is, the better the fatigue resistance performance of bitumen is, and the stronger its ability to resist the long-term deformation caused by fatigue is. The fatigue factor of five types of modified bitumen is shown in Figure 2. As can be seen from Figure 2, the main curves of the five modified bitumen are similar. With the gradual increase in temperature, the fatigue factors all gradually decrease. Although at 46 and 52 °C, the fatigue factor of PVC is higher, but at over 52 °C, the fatigue factor of PVC modified bitumen is lowest, which indicates that under high-temperature conditions, the fatigue resistance performance of PVC-modified bitumen is best, and its high-temperature resistance to fatigue is also stronger.

Based on the three sets of data of the complex modulus, phase angle, and fatigue factor, after unloading, the PS and PVC-modified bitumen have a better recovery performance than that of the other three types of modified bitumen, and the latter also has the best fatigue resistance property.

### 4.2. The Influence of Different Types of Waste Plastics on the Healing Ability of Bitumen

**Figure 3:**
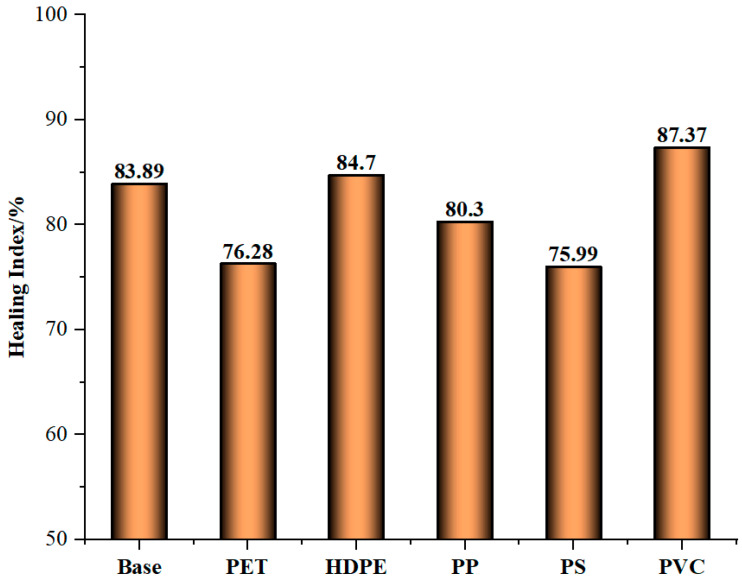
The healing index of five types of waste plastic-modified bitumen with 2% dosage.

Figure 3 shows the healing index calculated with a 4% dosage as an example. The healing index of PVC-modified bitumen is the highest, that is, its healing performance is the best. This is mainly because PVC undergoes physical grafting with bitumen, enhancing the structural strength and mechanical properties of bitumen, and thereby improving its self-healing ability [27]. The healing indices of the base and the bitumen modified by PET, HDPE, PP, PS, and PVC were 83.89%, 76.28%, 84.7%, 80.3%, 75.99%, and 87.37%, respectively. Compared with the healing index of the base bitumen, the healing indices of HDPE and PVC-modified bitumen increased by 0.81% and 3.84%, respectively. On the contrary, the healing indices of PET, PP, and PS-modified bitumen decreased by 7.61%, 3.59%, and 7.9%, respectively. Among the five modifiers, PVC has the most significant improvement on the healing performance of bitumen, followed by PP. This is because the dispersion of PVC in bitumen forms a microscopic network structure, which helps to disperse stress and reduce the formation and development of cracks [33]. The weakening of the healing performance of bitumen by HDPE is the most severe. This is because there is a significant difference between its molecular structure and that of bitumen, resulting in a weak interaction force, poor compatibility, and inability to be uniformly distribute in bitumen, leading to phase separation and thereby reducing the healing index [17].

### 4.3. The Influence of Different Dosages of Waste Plastics on the Healing Ability of Bitumen

**Figure 4:**
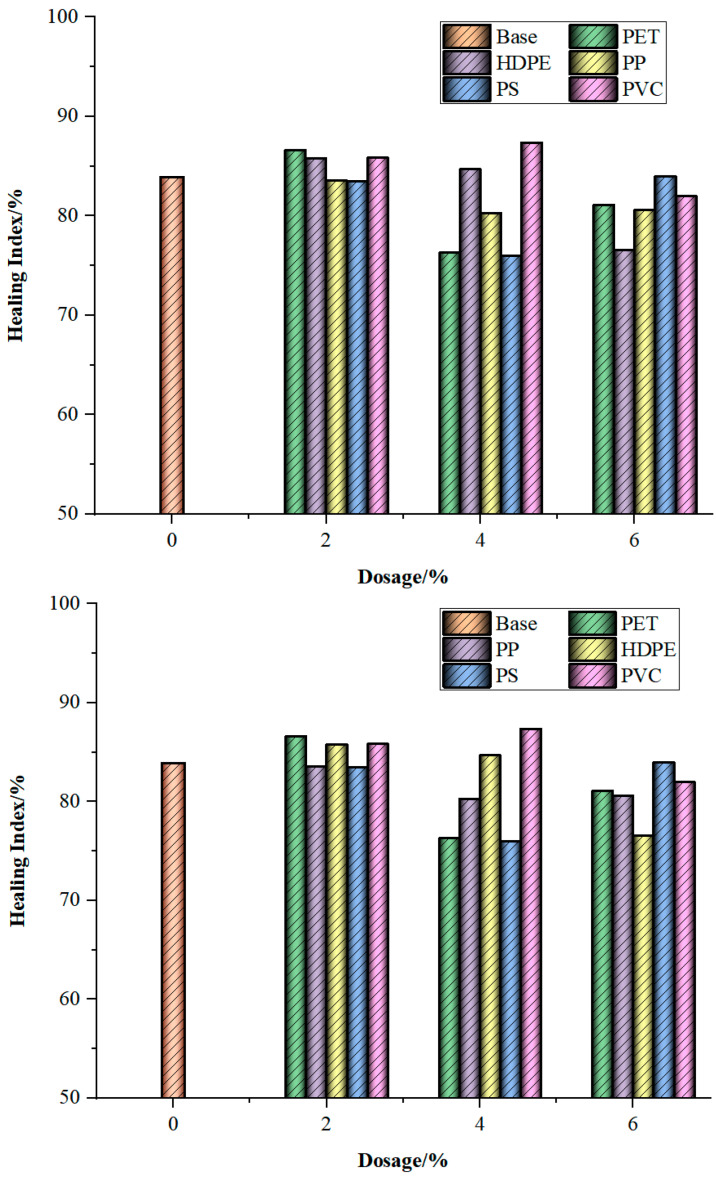
The healing index of five types of waste plastic-modified bitumen with different dosages.

As can be seen from Figure 4, the overall trend of the healing index change in the waste plastic-modified bitumen shows the characteristic of first increasing and then decreasing as the dosage of additives (PET, HDPE, PP, PS, and PVC) increases. After the initial fatigue loading, the healing index did not show a significant changing trend. However, after undergoing fatigue loading again, with the increase in the content of waste plastic, the healing index of PET, HDPE, and PP-modified bitumen reached the upper limit at 2%. The healing index of modified bitumen with PVC reached the upper limit at 4%. The reason is that the poor dispersion of waste plastic particles in bitumen and their large particle size lead to poor compatibility and low stability, which will introduce a large number of defects in bitumen. When the content of waste plastics is too high, it will cause problems such as stratification and segregation in the bitumen structure, and these phenomena will weaken the self-healing ability of the bitumen [34]. The healing index of the modified bitumen with PS reaches the upper limit at 6%. The upper limit value of the healing index of different modified bitumen (PET, HDPE, PP, PS, and PVC) increased by 2.69%, 1.92%, −0.33%, 0.05%, and 3.48%, respectively, compared with that of the base bitumen. Although the differences are small, there is evidence to prove that the addition of waste plastic breaks the original colloid structure of petroleum bitumen. The network-like structure formed by waste plastic restricts the activity of bitumen molecules and increases the viscoelasticity of bitumen, thereby reducing the sensitivity to temperature changes and improving the healing performance [35]. Overall, adding an appropriate amount of waste plastic can enhance the self-healing index of bitumen, but the proportion added must be precisely controlled. It is indicated that an appropriate amount of waste plastic promotes the improvement of the healing ability of bitumen and significantly enhances its fatigue resistance. To maximize the improvement effect of PET, HDPE, PP, PS, and PVC on the healing index of bitumen, the recommended optimal dosages of PET, HDPE, PP, PS, and PVC are 2%, 2%, 2%, 6%, and 4%, respectively.

From this, it can be known that bitumen pavement modified with waste plastic will show a stronger tolerance than traditional, ordinary bitumen when deformed. Especially under conditions of high temperatures and traffic congestion, plastic-modified bitumen can better resist fatigue and prevent crack propagation, demonstrating a superior crack resistance and durability.

### 4.4. The Influence of Multiple Damages on the Healing Ability of Waste Plastic-Modified Bitumen

**Figure 5:**
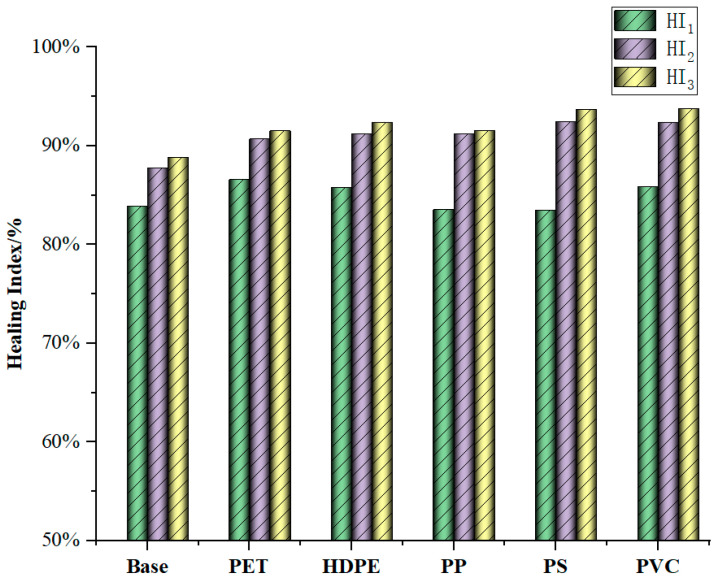
The healing index of five types of modified bitumen with waste plastics undergoing multiple damages.

Figure 5 shows the healing indicator calculated for five types of modified bitumen with 2% dosage additives and base bitumen as an example after undergoing multiple damages and healing repairs. As can be seen from Figure 5, during the first to second healing processes, the healing index of the waste plastic-modified bitumen generally showed an upward trend. The reasons for its growth are as follows: (1) in the experiment, there is a certain threshold for the degree of damage [36]. When the degree of damage of bitumen is lower than this threshold, it still has the ability to recover. After the intermittent period, the healing ability can be fully restored or increased. Due to the presence of modifiers, the threshold of the damage degree of modified bitumen has been further increased, enhancing its self-healing ability. (2) The cracks caused by multiple damages expand the mutual flow between the modifiers and bitumen molecules, promoting the adhesion between them, thereby increasing the self-healing ability of the crack surface [37]. (3) Under the action of modifiers, the deformation and recovery capacity of bitumen is modified, which increases its fatigue resistance and thereby enhances its self-healing ability.

Furthermore, it can also be seen from Figure 5 that although the HI_1_ value of some plastic-modified bitumen is lower than that of base bitumen (such as PP and PS-modified bitumen), after undergoing multiple damages, the improvement rate of the healing index of plastic-modified bitumen is much greater than that of base bitumen. The HI_2_ of base bitumen and five kinds of modified bitumen (PET, HDPE, PP, PS, and PVC) increased by 3.83%, 4.13%, 5.41%, 7.64%, 8.94%, and 6.55%, respectively, compared with HI_1_, in the order of PS > PP > PVC > HDPE > PET > base. While HI_3_ increased by 1.12%, 0.80%, 1.18%, 0.39%, 1.28%, and 1.42%, respectively, compared with HI_2_. The order is PVC > PS > HDPE > Base > PET > PP. Based on the above result, it can be known that the PS plastic modifier has better promoting effects on the damage healing ability of bitumen after undergoing multiple damages, while PET has the worst improvement effect. This is because the viscoelasticity of PS-modified bitumen is relatively high, and its sensitivity to temperature is relatively weak [24,26,38], which is consistent with the analysis results in Section 4.1 of this study. The phase angle of PET-modified bitumen is the highest (see Section 4.1), indicating that it has the worst elasticity and highest viscosity. During the long-term loading–healing process, its performance is reduced more and so is its damage healing ability for bitumen after undergoing multiple damages.

## 5. Conclusions

Multiple repeated loading tests with fatigue–healing–fatigue as the basic cycle were conducted on five modified bitumen containing different waste plastics (PET, HDPE, PP, PS, and PVC) with different dosages by a dynamic shear rheology (DSR) machine. The damage healing ability of bitumen of the same waste plastic with different dosage ratios and the same dosage of different waste plastics under the same healing time, loading strain, and damage degree through single and multiple loading was explored and analyzed. The conclusions are drawn as follows:

- PS and PVC-modified bitumen have a better recovery performance than that of the other three types of modified bitumen, and the latter also has the best fatigue resistance property.
- Among the five modifiers, PVC has the most significant improvement effect on the healing performance of bitumen, followed by PP. This is because the dispersion of PVC in bitumen forms a microscopic network structure, which helps to disperse stress and reduce the formation and development of cracks. The weakening of the healing performance of bitumen by HDPE is the most severe.
- Adding an appropriate amount of waste plastic can enhance the self-healing index of bitumen, but the proportion added must be precisely controlled. In order to maximize the improvement effect of PET, HDPE, PP, PS, and PVC on the healing index of bitumen, the recommended optimal dosages of PET, PP, HDPE, PS, and PVC are 2%, 2%, 2%, 6%, and 4%, respectively.
- Based on the multiple damage healing experiment, the result shows that the PS plastic modifier has the best promoting effect on the damage healing ability of bitumen after undergoing multiple damages, while PET has the worst improvement effect.

## Figures and Tables

**Table 1:** Technical index of 70 # base bitumen.

Technical Indicator	Soften Point/°C	Penetration/25 °C/0.1 mm	Ductility/15 °C
70 #	71.9	5.24	>100

**Table 2:** Density of plastic powder additives.

Additives	PET	HDPE	PP	PS	PVC
Apparent density (g/cm^3^)	1.375	0.95	0.958	1.047	1.335

## Data Availability

The raw data supporting the conclusions of this article will be made available by the authors on request.
